# Tyrosine hydroxylase conditional KO mice reveal peripheral tissue-dependent differences in dopamine biosynthetic pathways

**DOI:** 10.1016/j.jbc.2021.100544

**Published:** 2021-03-15

**Authors:** Katsuya Miyajima, Chiaki Kawamoto, Satoshi Hara, Masayo Mori-Kojima, Tamae Ohye, Chiho Sumi-Ichinose, Nae Saito, Toshikuni Sasaoka, Daniel Metzger, Hiroshi Ichinose

**Affiliations:** 1 School of Life Science and Technology, Tokyo Institute of Technology, Yokohama, Japan; 2 Department of Genetic Counseling, Graduate School of Health Sciences, Fujita Health University, Toyoake, Aichi, Japan; 3 Department of Pharmacology, School of Medicine, Fujita Health University, Toyoake, Aichi, Japan; 4 Department of Comparative and Experimental Medicine, Center for Bioresource-based Researches, Brain Research Institute, Niigata University, Niigata, Japan; 5 Department of Molecular and Cellular Medicine, Graduate School of Medical and Dental Sciences, Niigata University, Niigata, Japan; 6 Université de Strasbourg, Centre National de la Recherche Scientifique, UMR7104, Institut National de la Santé et de la Recherche Médicale, U1258, IGBMC, Illkirch, France

**Keywords:** adrenal, 3,4-dihydroxyphenylalanine, dopamine, gene KO, heart, noradrenaline, pancreas, stomach, sympathoadrenal system, tyrosine hydroxylase, AADC, aromatic amino acid decarboxylase, AD, adrenaline, APUD, amine precursor uptake and decarboxylation, CA, catecholamine, cKO, conditional KO, DA, dopamine, DBH, dopamine β-hydroxylase, DOPA, 3,4-dihydroxyphenylalanine, D1R, dopamine D1 receptor, LAT, L-type amino acid transporter, NA, noradrenaline, TAM, tamoxifen, TH, tyrosine hydroxylase

## Abstract

Dopamine (DA) exerts well-known functions in the brain as a neurotransmitter. In addition, it plays important physiological roles in peripheral organs, but it is largely unknown how and where peripheral DA is synthesized and regulated. Catecholamines in peripheral tissues are either produced within the tissue itself and/or derived from sympathetic neurons, which release neurotransmitters for uptake by peripheral tissues. To evaluate DA-producing ability of each peripheral tissue, we generated conditional KO mice (cKO mice) in which the tyrosine hydroxylase (TH) gene is ablated in the sympathoadrenal system, thus eliminating sympathetic neurons as a DA source. We then examined the alterations in the noradrenaline (NA), DA, and 3,4-dihydroxyphenylalanine (DOPA) contents in peripheral organs and performed immunohistochemical analyses of TH-expressing cells. In the heart and pancreas of cKO mice, both the TH protein and NA levels were significantly decreased, and the DA contents were decreased in parallel with NA contents, indicating that the DA supply originated from sympathetic neurons. We found TH-immunoreactive cells in the stomach and lung, where the TH protein showed a decreasing trend, but the DA levels were not decreased in cKO mice. Moreover, we found a significant correlation between the DA content in the kidney and the plasma DOPA concentration, suggesting that the kidney takes up DOPA from blood to make DA. The aforementioned data unravel differences in the DA biosynthetic pathway among tissues and support the role of sympathetic neurons as a DA supplier.

Dopamine (DA) is a neurotransmitter in the brain, and alterations in DA metabolism are related to the pathophysiology of neuropsychiatric disorders, such as Parkinson's disease, schizophrenia, and some developmental disorders. In addition to its role in the brain, DA also plays pivotal physiological functions in peripheral tissues, such as natriuresis in the kidney (1, 2, 3), glucose-stimulated insulin secretion in the pancreas (4, 5, 6, 7), liquid clearance in the lung (8), and acid secretion in the stomach (9, 10). DA regulates these functions *via* DA receptors belonging to G protein–coupled receptors. Despite the important physiological function, less attention has been given to the metabolism of DA in these peripheral organs.

Gastrointestinal dysfunction is prevalent in patients with Parkinson's disease, a dopaminergic neurodegenerative disease (11, 12). DA D2 receptor antagonists, antipsychotic drugs for schizophrenia, often present side effects, such as increasing insulin resistance and weight gain (13). As these symptoms are thought to be caused by perturbation of the peripheral DA metabolism, it is important for not only physiology but also pharmacology to clarify how peripheral DA concentration is regulated.

In the nervous system, DA is synthesized from tyrosine. Tyrosine hydroxylase (TH; enzyme commission number: 1.14.16.2) catalyzes the conversion of tyrosine to 3,4-dihydroxyphenylalanine (DOPA), the rate-limiting step to regulate the amount of DA (14). However, the synthesis and regulation of DA contents in peripheral tissues are largely unknown because of its complicated synthetic pathways.

There are three pathways to synthesize DA in peripheral tissues. A group of cells, called amine precursor uptake and decarboxylation (APUD) cells, produce DA from DOPA, which is taken up from the circulating blood, by the reaction of aromatic amino acid decarboxylase (AADC; enzyme commission number: 4.1.1.28), which catalyzes the conversion of DOPA to DA (15, 16). In contrast, the expression of TH has been reported in the pancreas (17, 18) and stomach (7, 19), suggesting the synthesis of DOPA by TH in these tissues. In addition, the sympathetic neurons innervating each organ can synthesize DA as a precursor of noradrenaline (NA) and may release DA under some circumstances (20). It is therefore difficult to know how much DA is intrinsically synthesized in each tissue, and whether the sympathetic neurons may contribute to local DA production in peripheral organs.

Although the circulating DOPA concentration may affect DA production in APUD cells, the origin of circulating DOPA also remains controversial. Previous reports have suggested that sympathetic neurons supply DOPA to the circulation (21). Another study indicated that sympathectomy through an abdominal incision did not affect muscle and arterial plasma DOPA levels (22).

To solve these issues, we conducted the present experiments to evaluate the intrinsically synthesized DA in each tissue after ablation of the *Th* gene in the sympathoadrenal system. We generated mice in which the *Th* gene can be ablated at adulthood in the sympathoadrenal system using a dopamine β-hydroxylase (DBH) promoter, which had been used for expression of transgenes in the sympathoadrenal system (23, 24). We assumed that if the nerve terminals of the sympathetic neurons are the major source of NA and DA in the tissue, the NA and DA contents should be decreased in parallel with the decreases in the TH protein levels in the mice. We also monitored the plasma DOPA levels because circulating DOPA can affect the DA production in APUD cells, and it is thought to be supplied from the sympathetic neurons (21). Furthermore, we performed immunohistochemical analyses and revealed that there were local TH- and AADC-expressing cells in the stomach and lung, whereas only AADC-expressing cells were present in the kidney. Our results clarified the systemic differences in the DA metabolism among peripheral tissues *in vivo* for the first time.

## Results

### Genetic ablation of the *TH* gene in sympathetic neurons and the adrenal gland

**Figure 1:**
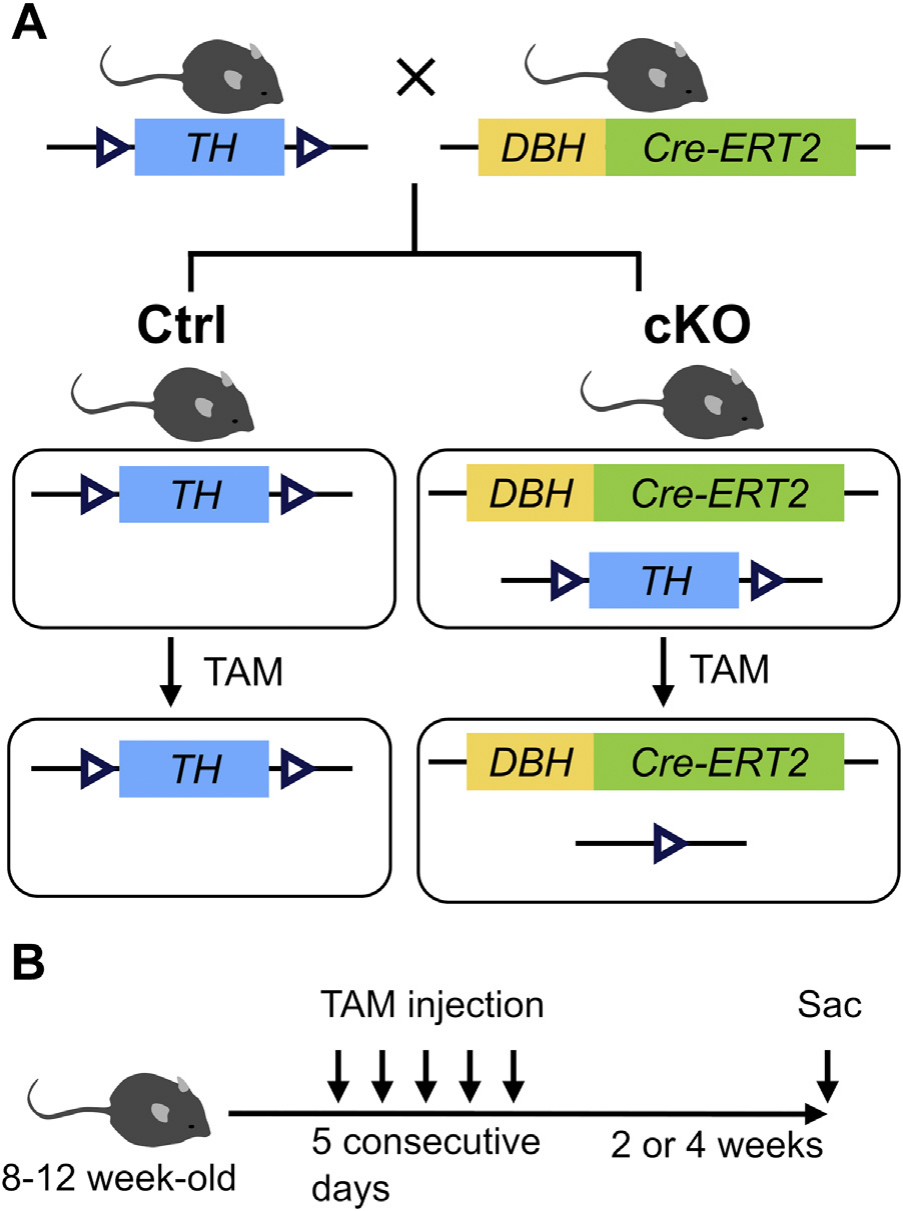
**Generation of sympathoadrenal-specific tyrosine hydroxylase (*Th*) KO mouse.***A*, schematic representation of the generation sympathoadrenal-specific *Th* KO mice. *Th*^*fl/fl*^ mice, in which exons 6 to 9 of the *Th* gene, are flanked with *loxP* sites (*open arrowhead*), were crossed with dopamine β-hydroxylase (*DBH*) *Cre-ERT2* mice, which express the Cre-ERT2 recombinase under the 5.8-kb promoter region of human *DBH* gene, to generate mice *Th*^fl/fl^ and *DBH Cre-ERT2* mice harboring loxP-flanked *Th* alleles. After administration of tamoxifen (TAM), the *Th* alleles are deleted in Cre-ERT2–expressing cells of *DBH Cre-ERT2*/*Th*^*fl/fl*^ mice (conditional KO [cKO]) but not of *Th*^*fl/fl*^ mice (Ctrl). *B*, schematic diagram of the experimental schedule. Eight- to 12-week-old male mice were intraperitoneally injected with 1 mg of TAM for 5 consecutive days. The mice were analyzed at 2 or 4 weeks after the last TAM injection.

DBH, the enzyme catalyzing the conversion of DA into NA, is selectively expressed in noradrenergic neurons and the adrenal medulla. To generate spatiotemporally-controlled somatic mutations of *Th* in DBH-expressing cells, we generated *DBH Cre-ERT2* mice that express the tamoxifen (TAM)-dependent Cre-ERT2 recombinase (25) under the control of the 5.8-kb promoter region of the human *DBH* gene and crossed them with floxed *Th* mice (*Th*^fl/fl^), in which exons 6 to 9 of the *Th* gene are flanked with *loxP* sites. Eight- to 12-week-old *Th*^fl/fl^ and *DBH Cre-ERT2*/*Th*^fl/fl^ mice were treated with TAM (Fujifilm-Wako) to generate Ctrl and conditional KO (cKO) mice (Fig. 1*A*), which were analyzed 2 or 4 weeks later (Fig. 1*B*).

**Figure 2:**
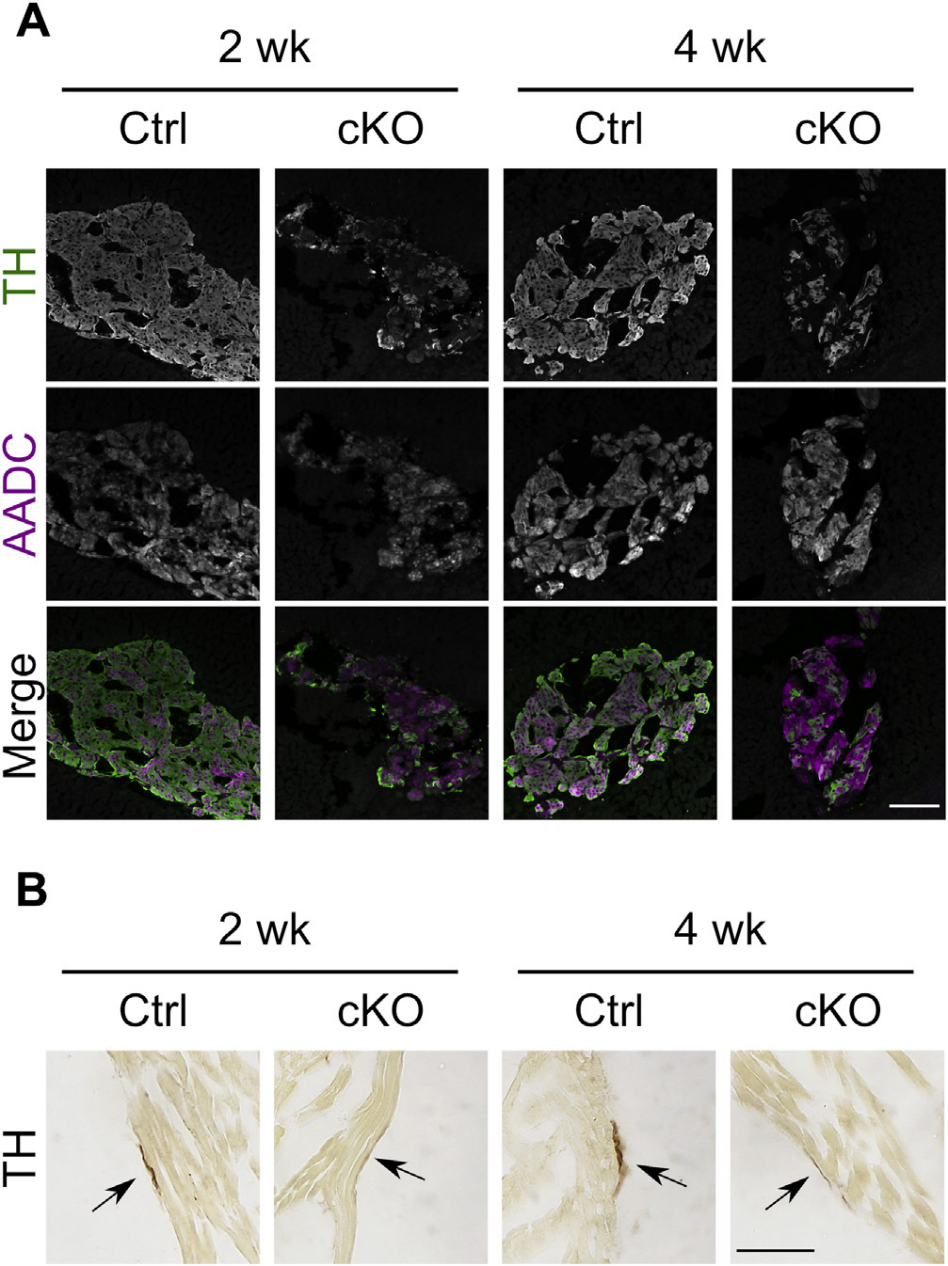
**Immunohistochemical analysis of the adrenal gland and heart after administration of tamoxifen (TAM).***A*, tissue sections of the adrenal gland of *Th*^fl/fl^ (Ctrl) and dopamine β-hydroxylase *Cre-ERT2/Th*^fl/fl^ (conditional KO [cKO]) mice were prepared after 2 or 4 weeks after TAM injection (2 and 4 weeks, respectively). Tyrosine hydroxylase (TH) and aromatic amino acid decarboxylase (AADC) were visualized by immunofluorescent staining. Merged images were pseudocolored with TH (*green*) and AADC (*magenta*). Immunoreactivity of TH was decreased in slices of the adrenal gland from cKO mice compared with those of Ctrl mice. The scale bar represents 100 μm. *B*, tissue sections of the heart from Ctrl and cKO mice after 2 or 4 weeks after TAM injection. TH-positive nerve bundles (indicated by an *arrow*) in the surface of the heart from cKO mice were thinner than those of Ctrl mice. The scale bar represents 50 μm.

The TH expression was analyzed by immunohistochemistry. Whereas most cells were TH positive in the adrenal medulla of Ctrl mice, few TH-positive cells were detected in cKO mice at both 2 and 4 weeks after TAM injection (Fig. 2*A*). In contrast, AADC immunoreactivity did not decline, indicating that TH is selectively ablated in the adrenal medulla. We further examined the alteration in the TH protein in the sympathetic neurons of the heart (Fig. 2*B*). The TH-positive nerve bundle was thinner in the cKO mice than in Ctrl mice (Fig. 2*B*, *arrow*). In addition, we confirmed that the TH protein levels were not affected in cKO mice before TAM injection by Western blot analysis (Fig. S1*A*). To confirm tissue-specific recombination in cKO mice, we examined *Th* recombination in tissues at 4 weeks after TAM injection by PCR. As shown in Figure S1*B*, recombined *Th* alleles were detected in the DNAs extracted from the adrenal gland and superior cervical ganglion, where the sympathetic neuronal soma are located. Such alleles were also detected in the lung and weakly in the heart. Immunostaining of lung sections with anti-TH antibodies revealed similar patterns in cKO and Ctrl mice, showing that TH-positive cells in the lung were not affected in cKO mice (Fig. S1*C*). Thus, we successfully generated mice in which the *Th* gene is selectively ablated in the sympathoadrenal system after TAM injection.

### Alterations in TH protein and catecholamine levels in the adrenal gland

**Figure 3:**
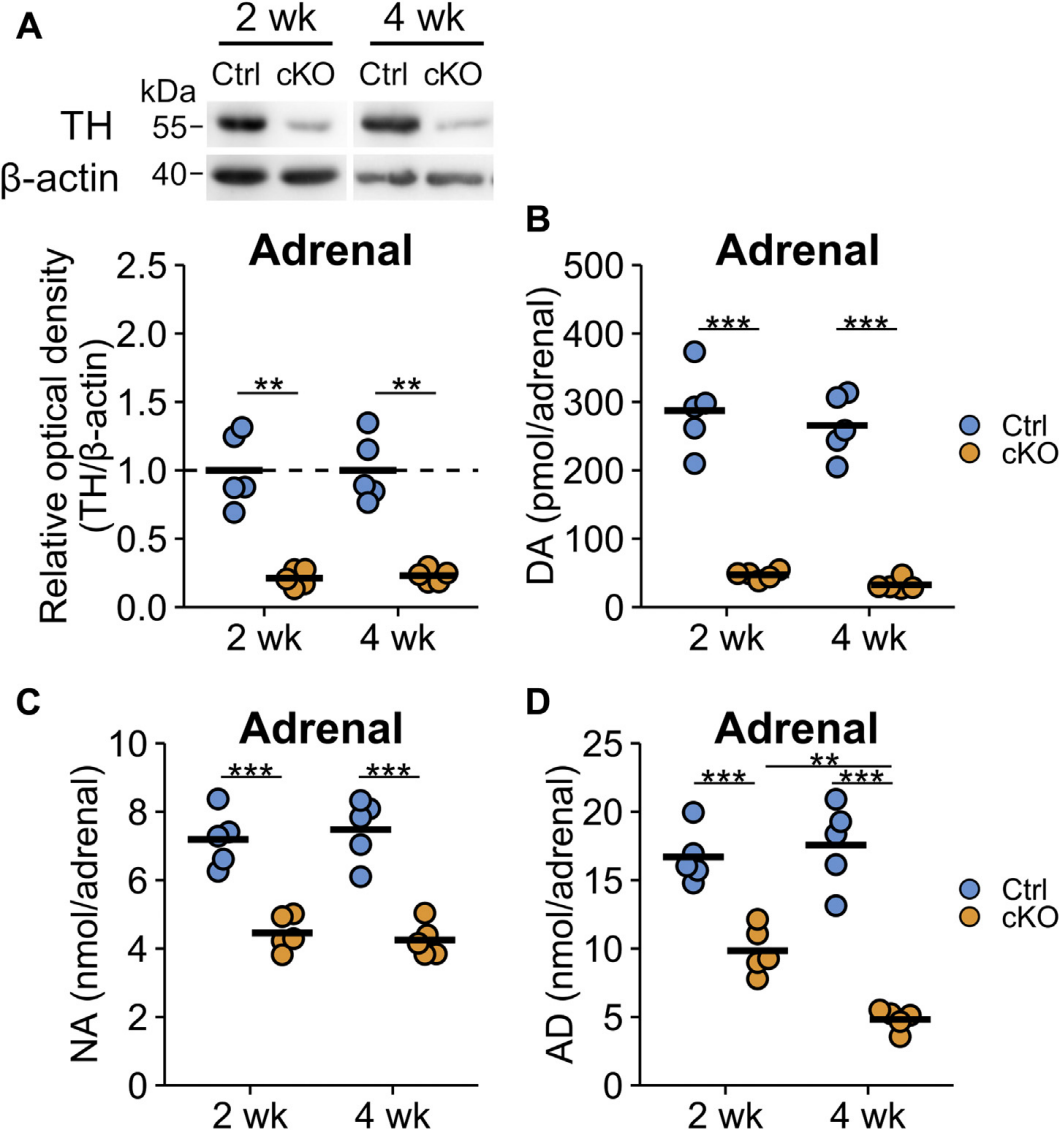
**Tyrosine hydroxylase (TH) protein and catecholamine levels in the adrenal gland were decreased in the conditional KO (cKO) mice.***A*, adrenal glands of *Th*^fl/fl^ (Ctrl) and dopamine β-hydroxylase *Cre-ERT2/Th*^fl/fl^ (cKO) mice were subjected to the Western blot analysis after 2 or 4 weeks after tamoxifen injection (2 and 4 weeks, respectively). The relative band intensities of TH were represented as the ratio of Ctrl mice in 2 and 4 weeks, separately. The *blue circles* indicate the values of Ctrl mouse, *orange circles* indicate those of cKO mouse, and the bars indicate the means. n = 5 in each group. ∗∗*p* < 0.01, Welch's two-tailed *t* test with Holm's correction. *B*, the dopamine (DA) contents in the adrenal gland of Ctrl and cKO mice. *C*, the noradrenaline (NA) contents in the adrenal gland of Ctrl and cKO mice. *D*, the adrenaline (AD) contents in the adrenal gland of Ctrl and cKO mice. The *blue circles* indicate Ctrl mouse, *orange circles* indicate cKO mouse, and the bars indicate the means. n = 5 in each group. ∗∗*p* < 0.01; ∗∗∗*p* < 0.001, Tukey–Kramer test.

We next examined the TH protein levels by Western blot analyses and measured the catecholamine (CA) contents in the tissues at 2 or 4 weeks after TAM injection. The TH levels in the adrenal gland were fivefold lower in cKO mice than in Ctrl mice at both 2 and 4 weeks after TAM injection (Fig. 3*A*). CA levels in the adrenal glands of cKO mice showed distinct profiles. DA in cKO mice was decreased to approximately 15% compared with Ctrl mice at both 2 and 4 weeks (287 ± 27 *versus* 47 ± 3 pmol/adrenal gland at 2 weeks [*p* < 0.001] and 265 ± 20 *versus* 33 ± 4 pmol/adrenal gland at 4 weeks [*p* < 0.001] in Ctrl and cKO mice, respectively) (Fig. 3*B*). Because DA is an intermediate metabolite that produces NA and adrenaline (AD) in the adrenal gland, the parallel decreases in the DA contents with those of TH protein indicated that the TH activity in the adrenal gland was decreased to 15 to 20% of Ctrl in cKO mice. In contrast, the decreases in NA levels were only approximately 60% at both 2 and 4 weeks (7.19 ± 0.36 *versus* 4.46 ± 0.23 nmol/adrenal gland at 2 weeks [*p* < 0.001] and 7.48 ± 0.41 *versus* 4.25 ± 0.22 nmol/adrenal gland at 4 weeks [*p* < 0.001] in Ctrl and cKO mice, respectively) (Fig. 3*C*), and the AD level in cKO mice was 59% of that in Ctrl mice at 2 weeks and further decreased to 27% at 4 weeks (16.68 ± 0.89 *versus* 9.84 ± 0.78 nmol/adrenal gland at 2 weeks [*p* < 0.001] and 17.56 ± 1.35 *versus* 4.82 ± 0.34 nmol/adrenal at 4 weeks [*p* < 0.001] in Ctrl and cKO mice, respectively) (Fig. 3*D*).

### Plasma CA contents

**Figure 4:**
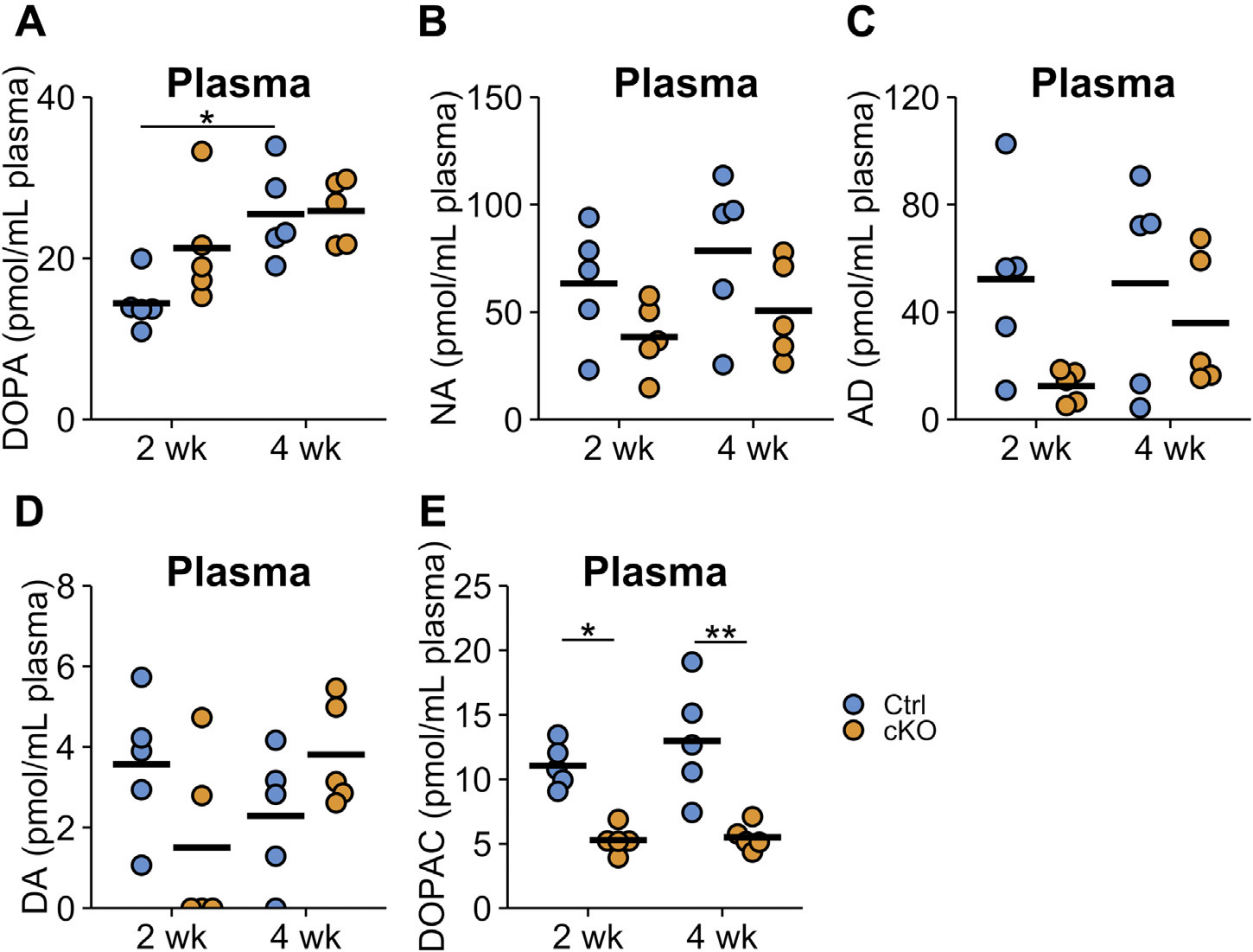
**Catecholamine contents in the plasma of Ctrl and conditional KO (cKO) mice.** Summary of the catecholamine contents in the plasma of *Th*^fl/fl^ (Ctrl) and dopamine β-hydroxylase *Cre-ERT2/Th*^fl/fl^ (cKO) at 2 or 4 weeks after tamoxifen injection (2 and 4 weeks, respectively). (*A*) dihydroxyphenylalanine (DOPA), (*B*) noradrenaline (NA), (*C*) adrenaline (AD), (*D*) dopamine (DA), and (*E*) dihydroxyphenylacetic acid (DOPAC) were measured by HPLC-electrochemical detection. The *blue circles* indicate Ctrl mouse, *orange circles* indicate cKO mouse, and the bars indicate the means. n = 5 in each group. ∗*p* < 0.05, Tukey–Kramer test.

The aforementioned experiments show that the *TH* gene was deleted in sympathetic noradrenergic neurons and the adrenal medulla after TAM injection. Although we presumed that sympathetic neurons release DOPA into the circulation, the plasma DOPA levels of cKO mice were comparable to those of Ctrl mice at both 2 and 4 weeks after TAM injection (Fig. 4*A*). This result indicates that sympathetic neurons are not the main source of DOPA in plasma.

In contrast, the mean NA and AD contents in plasma of cKO mice were decreased to approximately 20 to 70% compared with those of Ctrl mice, but these differences were not statistically significant because of large variation (Fig. 4, *B* and *C*). These plasma CA reductions may reflect the reduction of TH activity in the sympathoadrenal system.

The concentrations of DA in plasma were one-tenth of those of NA and AD and below the detection limit for some samples (Fig. 4*D*). Even though we did not observe a significant alteration in the DA content in cKO mice, the levels of dihydroxyphenylacetic acid, a metabolite of DA, were significantly decreased in cKO mice to approximately 50% compared with Ctrl mice (11.05 ± 0.77 *versus* 5.28 ± 0.47 pmol/ml plasma at 2 weeks [*p* = 0.0103] and 12.98 ± 1.99 *versus* 5.5 ± 0.46 pmol/ml plasma at 4 weeks [*p* = 0.0011] in Ctrl and cKO mice, respectively) (Fig. 4*E*).

### Alterations in TH protein and NA levels in peripheral tissues

**Figure 5:**
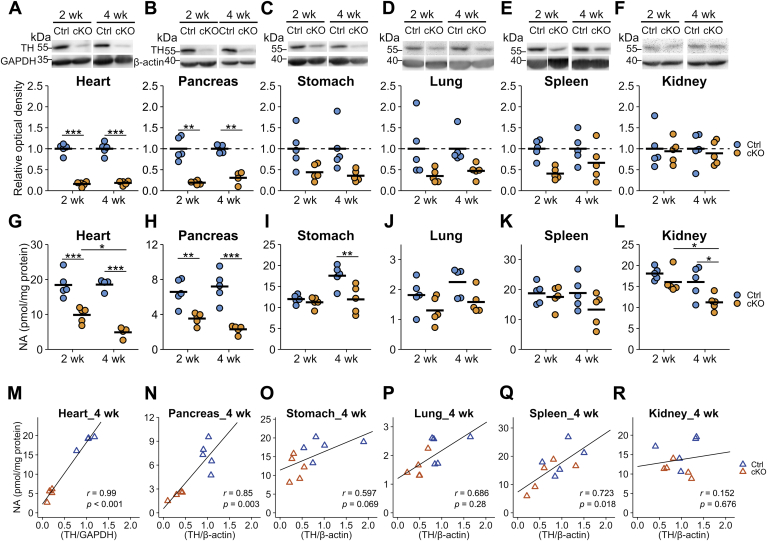
**Alterations in the tyrosine hydroxylase (TH) protein contents and the levels of noradrenaline in the tissues.** Tissue homogenates were subjected to Western blot analysis for quantitation of the TH protein and β-actin in the *Th*^fl/fl^ (Ctrl) and dopamine β-hydroxylase *Cre-ERT2/Th*^fl/fl^ (conditional KO [cKO]) at 2 or 4 weeks after tamoxifen injection (2 and 4 weeks, respectively). GAPDH was used as a loading control. *A*–*F*, quantitation of the TH protein in the heart (*A*), pancreas (*B*), stomach (*C*), lung (*D*), spleen (*E*), and kidney (*F*). The *blue circles* indicate Ctrl mouse, *orange circles* indicate cKO mouse, and the *bars* indicate the means. The band intensities of TH were represented as the ratio of Ctrl mice in 2 and 4 weeks, separately. n = 5 in each group, except for Ctrl and cKO at 4 weeks of heart, n = 4. ∗∗*p* < 0.01; ∗∗∗*p* < 0.05, Welch's two-tailed *t* test corrected by Holm's method. *G*–*L*, the contents of noradrenaline (NA) in the heart (*G*), pancreas (*H*), stomach (*I*), lung (*J*), spleen (*K*), and kidney (*L*). n = 5 in each group, except for Ctrl and cKO at 4 weeks of heart, n = 4. ∗*p* < 0.05; ∗∗*p* < 0.01; ∗∗∗*p* < 0.001, Tukey–Kramer test. *M*–*R*, the correlations between the NA contents and the TH protein amounts in the heart (*M*), pancreas (*N*), stomach (*O*), lung (*P*), spleen (*Q*), and kidney (*R*). The *y*-axis represents the NA contents, and the *x*-axis does the TH contents. *Lines* indicate the linear fitting, *blue triangles* indicate Ctrl mouse, and *orange triangles* indicate cKO mouse. Pearson's correlation coefficients were shown in the *plots*.

The TH protein levels in heart and pancreas of cKO mice were significantly decreased to approximately 20% compared with Ctrl mice at both 2 and 4 weeks after TAM injection (Fig. 5, *A* and *B*). They were decreased to approximately 40 to 60% in the stomach, lung, and spleen of cKO mice compared with that in Ctrl mice, even though it did not reach statistical significance (Fig. 5, *C*–*E*), and unaffected in the kidney (Fig. 5*F*).

The NA contents in the heart and pancreas were significantly reduced in cKO mice compared with Ctrl mice to approximately 30 to 50%, correlating with the decreases in the TH protein levels (Fig. 5, *G* and *H*). In other tissues, there was little or no decline in the NA levels in cKO mice, whereas the NA levels in the stomach and kidney at 4 weeks after TAM injection were reduced to approximately 70% of those in Ctrl mice (Fig. 5, *I*–*L*).

By plotting the NA levels against the TH protein levels (Fig. 5, *M*–*R*), a strong correlation of the NA and TH protein levels was observed in the heart and pancreas (*r* = 0.99, *p* < 0.001 in the heart; and *r* = 0.85, *p* = 0.003 in the pancreas; Fig. 5, *M* and *N*), a weak correlation in the stomach, lung, and spleen (*r* = 0.597, *p* = 0.069 in the stomach; *r* = 0.686, *p* = 0.285 in the lung; and *r* = 0.723, *p* = 0.018 in the spleen; Fig. 5, *O*–*Q*), and no correlation in the kidney (*r* = 0.152, *p* = 0.676; Fig. 5*R*). The good correlation between the NA and TH protein levels in the heart and pancreas suggests that most of the TH protein and NA in the tissues are derived from the nerve terminals of the sympathetic neurons. In other tissues, as only a weak or no correlation between the TH and NA levels was observed, it suggests the presence of endogenous TH-expressing cells and/or cells taking up and storing NA from circulating blood through NA transporters in these organs.

### DOPA and DA contents in peripheral organs

**Figure 6:**
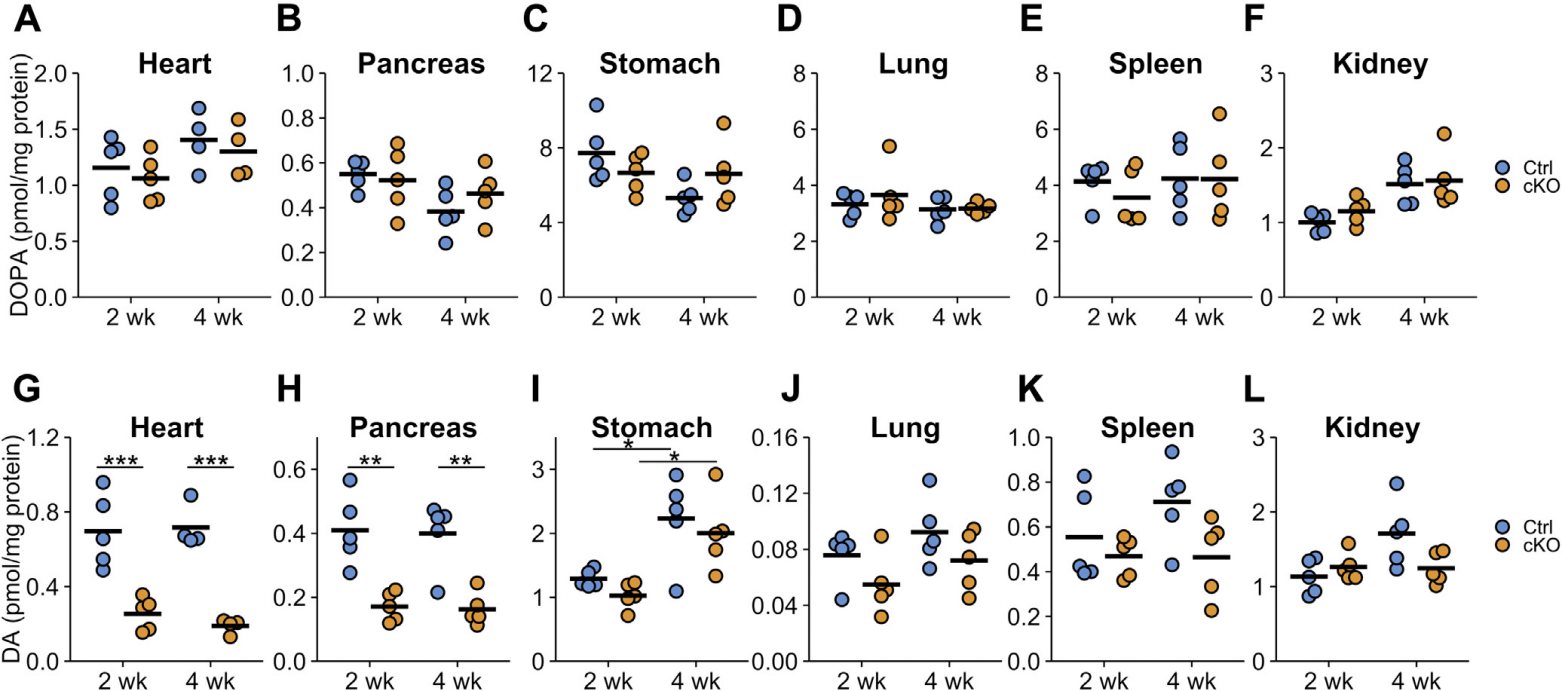
**Dihydroxyphenylalanine (DOPA) and dopamine contents after tamoxifen (TAM) injection to conditional KO (cKO) mouse.***A*–*F*, the contents of DOPA in the heart (*A*), pancreas (*B*), stomach (*C*), lung (*D*), spleen (*E*), and kidney (*F*) of *Th*^fl/fl^ (Ctrl) and dopamine β-hydroxylase *Cre-ERT2/Th*^fl/fl^ (cKO) mice at 2 or 4 weeks after TAM injection (2 and 4 weeks, respectively). The *blue circles* indicate Ctrl mouse, *orange circles* indicate cKO mouse, and the bars indicate the means. There were no statistical differences in a Tukey–Kramer test. *G*–*L*, the contents of dopamine (DA) in the heart (*G*), pancreas (*H*), stomach (*I*), lung (*J*), spleen (*K*), and kidney (*L*) of Ctrl and cKO mice at 2 or 4 weeks after TAM injection. n = 5 in each group, except for Ctrl and cKO at 4 weeks of heart, n = 4. ∗*p* < 0.05; ∗∗*p* < 0.01; ∗∗∗*p* < 0.001, Tukey–Kramer test.

Figure 6 summarizes the DOPA and DA contents in the peripheral organs. DOPA concentrations were not decreased after TAM injection in the examined tissues (Fig. 6, *A*–*F*). However, the contents of DOPA varied between organs: approximately 4 to 8 pmol/mg protein in the stomach, lung, and spleen, and approximately 1 pmol/mg protein in the heart, pancreas, and kidney (Fig. 6, *A*–*F*), indicating that the tissue DOPA levels were controlled by mechanisms other than the concentration of DOPA in the plasma.

DA contents in the heart and pancreas of cKO mice were decreased to approximately 30 and 40% of Ctrl mice, respectively (Fig. 6, *G* and *H*), but the DA contents in the kidney, lung, spleen, and stomach of cKO mice showed less or no change in comparison to Ctrl mice (Fig. 6, *I*–*L*). These data suggest that DA in the heart and pancreas is mainly derived from sympathetic neurons, whereas DA in the kidney, lung, spleen, and stomach is derived from DOPA in the plasma and/or from DOPA synthesized by TH-expressing cells in the organ.

### Correlations between DA and NA as well as tissue DOPA or DA and plasma DOPA

**Figure 7:**
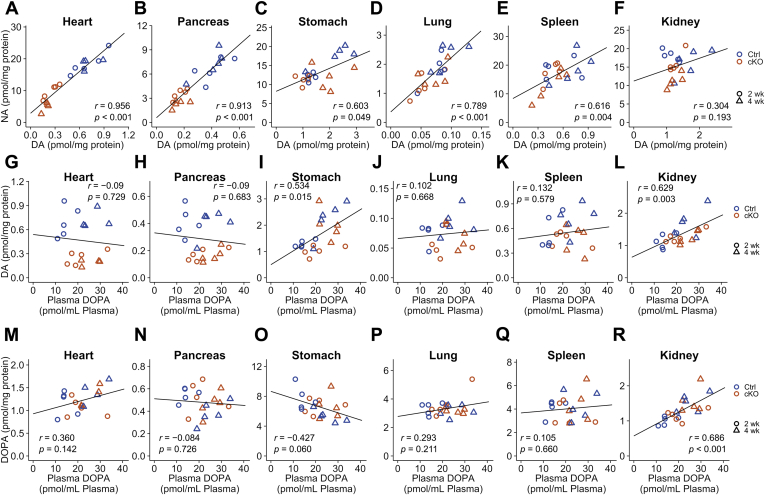
**Correlations between tissue dopamine (DA) and noradrenaline (NA) levels, between tissue DA and plasma 3,4-dihydroxyphenylalanine (DOPA) levels, and between tissue DOPA and plasma DOPA levels.***A*–*F*, correlations between NA and DA levels in the heart (*A*), pancreas (*B*), stomach (*C*), lung (*D*), spleen (*E*), and kidney (*F*) of *Th*^fl/fl^ (Ctrl) and dopamine β-hydroxylase *Cre-ERT2/Th*^fl/fl^ (conditional KO [cKO]) at 2 or 4 weeks after tamoxifen injection. The *y*-axis represents the NA content, and the *x*-axis represents DA content, and the lines indicate the linear fitting. *G*–*L*, correlations between plasma DOPA and tissue DA levels in the heart (*G*), pancreas (*H*), stomach (*I*), lung (*J*), spleen (*K*), and kidney (*L*) of the Ctrl and cKO mice at 2 and 4 weeks. The *y*-axis represents DA contents, and the *x*-axis represents plasma DOPA levels. Lines indicate the linear fitting. *M*–*R*, correlations between plasma DOPA and tissue DOPA levels in the heart (*M*), pancreas (*N*), stomach (*O*), lung (*P*), spleen (*Q*), and kidney (*R*) of Ctrl and cKO mice at 2 and 4 weeks after tamoxifen injection. The *y*-axis represents DOPA contents, and the *x*-axis represents plasma DOPA levels. Lines indicate the linear fitting. *Blue-colored points* indicate Ctrl mice, *orange-colored points* indicate cKO mice, *circle-shaped points* indicate the 2 weeks, and *triangle-shaped points* indicate the 4 weeks. Pearson's correlation coefficients were shown in the *plots*.

We plotted the tissue NA levels against the tissue DA levels (Fig. 7, *A*–*F*). DA in peripheral tissues corresponds to DA synthesized in sympathetic neurons and in the endogenous tissue DA. Because we genetically ablated *Th* in sympathetic neurons, DA was considered to be synthesized in endogenous cells and sympathetic neurons when the correlation between NA and DA was low. As shown in Figure 7, NA and DA in the heart, pancreas, and lung showed strong correlations (*r* = 0.956, *p* < 0.001 in the heart; *r* = 0.913, *p* < 0.001 in the pancreas; and *r* = 0.789, *p* < 0.001 in the lung; Fig. 7, *A*, *B*, and *D*). Moreover, NA and DA in the stomach and spleen showed moderate correlations (*r* = 0.603, *p* = 0.005 in the stomach; and *r* = 0.616, *p* = 0.004 in the spleen; Fig. 7, *C* and *E*), and NA and DA in the kidney showed weak correlations (*r* = 0.304, *p* = 0.193; Fig. 7*F*). These results indicate that DA is not mainly synthesized in the sympathetic neurons in the stomach, spleen, and kidney.

We analyzed the correlation of tissue DOPA and DA with plasma DOPA to confirm whether tissue DA was dependent on the levels of DOPA in the plasma (Fig. 7, *G*–*R*). There were moderate correlations in the kidney and stomach (*r* = 0.629, *p* = 0.003 in the kidney and *r* = 0.534, *p* = 0.015 in the stomach; Fig. 7, *I* and *L*), whereas no correlation was observed in the heart, pancreas, lung, and spleen (Fig. 7, *G*, *H*, *J*, and *K*). In addition, the correlation of tissue DOPA and plasma DOPA in each organ was tested. Only the kidney showed a strong correlation (*r* = 0.686, *p* < 0.001; Fig. 7*R*). The significant correlation of the DOPA and DA contents in the kidney with the DOPA concentration in the plasma suggests that DA in the kidney is derived from circulating DOPA.

### TH and AADC localization in dopamine D1 receptor reporter mice

Finally, we investigated the distribution of TH and AADC by immunohistochemistry in the stomach, lung, and kidney, which showed less correlation of DA and NA (Fig. 7, *C*, *D*, and *F*), to evaluate whether DA can be synthesized from tyrosine and/or circulating DOPA. Because the dopamine D1 receptor (D1R) plays an important role in DA function in these peripheral tissues (10, 26, 27, 28, 29), we used D1R reporter mice, which express β-galactosidase under the D1R promoter (30), to visualize D1R-expressing cells by X-gal staining.

**Figure 8:**
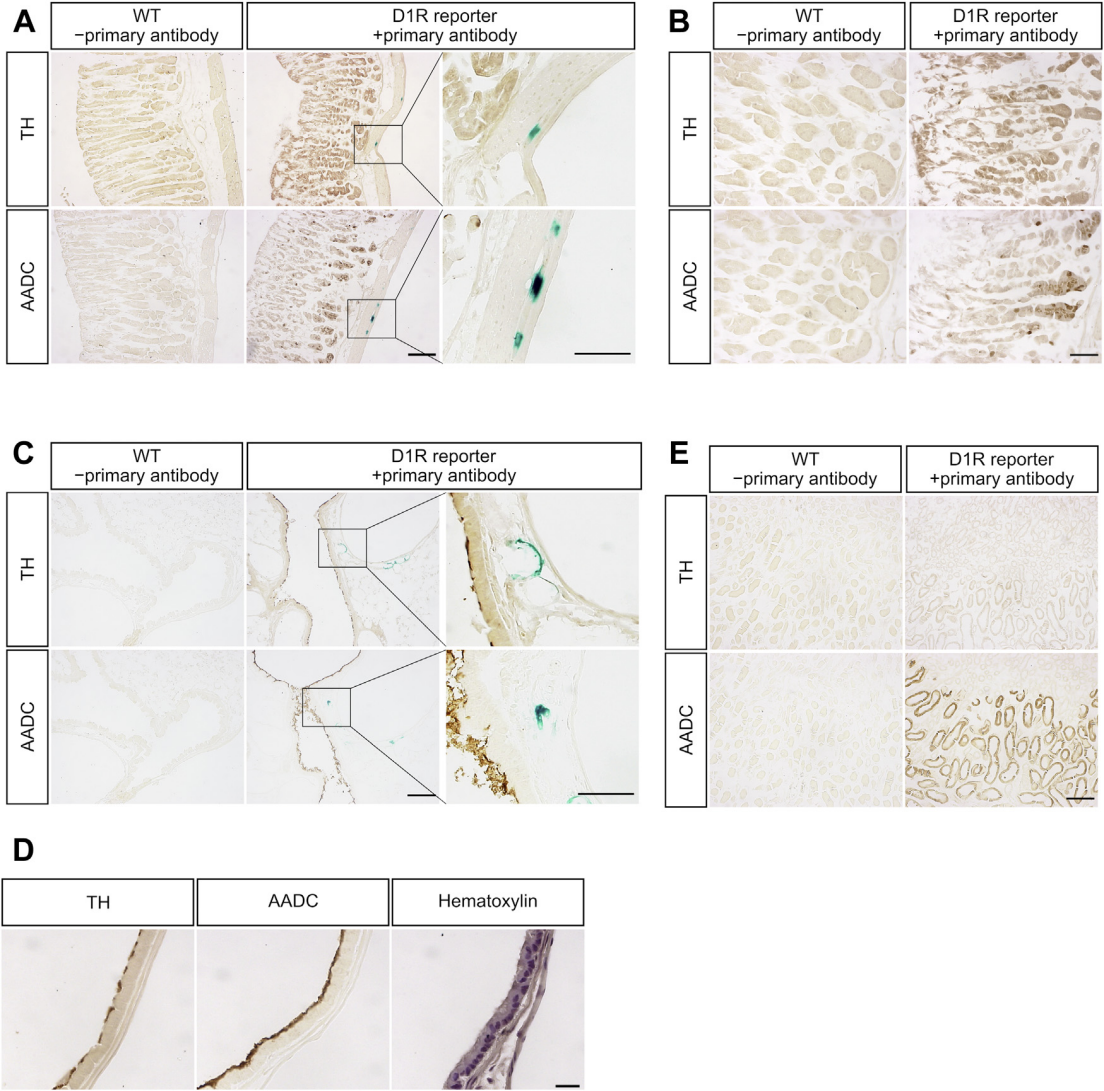
**Immunohistochemical analysis of tyrosine hydroxylase (TH) and aromatic amino acid decarboxylase (AADC) in the stomach, lung, and kidney and expression of dopamine D1 receptor (D1R).***A*, TH and AADC were immunostained after X-gal staining in the corpus of stomach of WT mouse and D1R reporter mouse. Tissue slices of the WT mouse were incubated without primary antibody for negative control. TH and AADC were expressed in the mucosa of stomach. X-gal staining was only detected in the muscularis externa of stomach, showing higher magnification images in the third column. The *scale bars* represent 100 μm in the first and second columns and 20 μm in the third column. *B*, higher magnification images of the tissue slice are shown in *A*. TH and AADC were immunostained after X-gal staining in the stomach of WT mouse and D1R reporter mouse. Tissue slices of the WT mouse were incubated without primary antibody for negative control. The scale bar represents 50 μm. *C*, TH and AADC were immunostained after X-gal staining in the lung of WT mouse and D1R reporter mouse. The tissue slices of the WT mouse were incubated without primary antibody for negative control. TH and AADC immunoreactivities were detected in the airway epithelium cells. X-gal staining was detected near the TH- and AADC-positive cells but not in the same cells. The *scale bars* represent 100 μm in the first and second columns and 20 μm in the third column. *D*, higher magnification images of the tissue slice are shown in *C*. TH, AADC, and hematoxylin staining are shown. TH and AADC immunoreactivities were detected in the surface of airway epithelial cells. The scale bar represents 50 μm. *E*, TH and AADC were immunostained after X-gal staining in the kidney of WT and D1R reporter mouse. Tissue slices of the WT mouse were incubated without primary antibody for negative control. AADC was strongly detected in the outer medulla, whereas TH was not detected. X-gal staining was not detected in the slices examined. The *scale bar* represents 100 μm.

TH and AADC were immunodetected in the corpus mucosa of the stomach; however, X-gal staining was sparsely detected in the muscularis externa but not in TH- and AADC-positive cells (Fig. 8, *A* and *B*). D1R-positive cells in the muscularis externa may be involved in gastric motility, which has been reported to be modulated by DA (31); however, the source of DA may not be endogenous DA-producing cells but another cell, *e.g.*, sympathetic neurons. In the lung, immunoreactivities of TH and AADC were detected in the epithelial cells of the airway. However, X-gal staining was not detected in TH- and AADC-immunoreactive cells, but it was observed around the blood vessels (Fig. 8*C*). TH and AADC were localized on the cell surface of airway epithelial cells compared with the counterstained slices with hematoxylin (Fig. 8*D*). In the kidney, TH was not stained, whereas AADC was immunoreactive in the outer medulla (Fig. 8*E*). No X-gal staining was detected in either the cortex or medulla of the kidney.

## Discussion

In this study, we examined alterations in TH expression and quantified the concentrations of DOPA, DA, and NA in the heart, pancreas, stomach, lung, spleen, and kidney of mice in which the *Th* gene was selectively ablated in the sympathoadrenal system.

It is striking that ablation of TH in the adrenal gland affects DA much more than NA and AD (Fig. 3). NA and AD are stored in chromaffin granules and large intracellular vesicles, and NA and AD can be recycled from circulating blood through the NA transporters SLC6A2 (32, 33). Although DA is also a substrate of the NA transporter, circulating DA concentrations are less than one-tenth of those of NA and AD (Fig. 4). Therefore, the slower declines in the NA and AD levels may be attributed to the action of the NA transporter and might account for the slower turnover rate of these CAs in the adrenal gland (34).

Plasma DOPA levels were not reduced at either 2 or 4 weeks after TAM injection, although the TH protein levels were greatly decreased in the sympathoadrenal system (Fig. 4*A*). These results suggest that the sympathetic nervous system and adrenal medulla should not be the direct sources of DOPA in the plasma. Previous reports have suggested that sympathetic neurons supply DOPA to the circulation by the experiments of 6-hydroxydopamine administration (21) and immobilization stress (35). Consistent with our results, sympathectomy through an abdominal incision did not affect muscle and arterial plasma DOPA levels (22). Stress responses of the sympathoadrenal system may trigger DOPA production in other cells.

The mice analyzed in this experiment did not exhibit weight loss and abnormal appearance up to 4 weeks after TAM administration. Our preliminary experiments showed that blood pressure, heart rate, and distance traveled in open field test were similar in control and cKO mice at 2 weeks after TAM injection. The defects in the present animal model might be milder than in other models produced by surgical and chemical sympathectomy, as the TH proteins were gradually decreased over 2 weeks to about 20% of the original levels (Figs. 3 and 5). The remaining sympathetic neurons may be able to compensate the function. However, the characterization of the mutant mice enabled to evaluate the precise biochemical alterations in each tissue. The effects of the decreased sympathetic activity on immune function and peripheral DA actions and phenotypes at later time in cKO mice remain to be evaluated.

TH reduction in cKO mice was lower in the stomach compared with those in the heart and pancreas (50% in the stomach and 20% in the heart and pancreas), and the correlation of NA and DA in the stomach was also less than that in the heart and pancreas (*r* = 0.603 in the stomach and *r* > 0.9 in the heart and pancreas). Interestingly, we show that TH is expressed in stomach mucosal cells (Fig. 8, *A* and *B*). Thus, our present data support the presence of a DOPA-synthesizing mechanism in stomach mucosal cells. Consistent with our results, it has previously been reported that TH is expressed in gastric mucosal cells (7, 10, 19). In addition, after ingestion of a standard mixed meal, the increment of plasma DOPA levels in humans with a Roux-en-Y gastric bypass surgery is reduced compared with that of control patients (7), suggesting that the stomach is a source of plasma DOPA.

DA in the adult pancreas has a physiological function to suppress glucose-stimulated insulin secretion. DA in β-cells is reported to be synthesized from extracellular DOPA by AADC (4, 36). However, a recent study has revealed differential TH expression among mice strains, that is, ∼35-fold more TH-positive β-cells in islets of PWK/PhJ and CAST/EiJ mice than of C57BL/6J mice (37). In the present study, we observed a strong correlation between TH and NA (*r* = 0.85) as well as between NA and DA (*r* = 0.913), indicating that most DA in the pancreas is derived from the sympathetic neurons. This result is consistent with the report that the DA content in β-cells synthesized from circulating DOPA is maintained at low levels (38). In combination with the present results, we postulate that the endogenous TH activity in the pancreas may be low compared with that in the sympathetic neurons, at least in C57BL/6J mice, while detailed studies are required to confirm that TH is involved in DA synthesis in β-cells.

DA receptors are expressed in the heart (39, 40), but the source of DA is not clear. One possible hypothesis is that sympathetic noradrenergic neurons release DA. Recent reports showed that optogenetic activation of noradrenergic neurons in the hippocampus slices augmented extracellular DA concentration as well as NA concentration (41). Thus, we consider that sympathetic neurons can also release DA and modulate tissue function *via* the DA receptor. In fact, it was reported that DA was not detected in the perfusate from the rat heart, whereas DA concentration was increased after 20 min of ischemia, which stimulate NA release from sympathetic neurons (20). Moreover, nepicastat, a selective DBH inhibitor, caused an increase in the DA contents as well as a decrease in the NA contents in the artery, left ventricle, and cerebral cortex (42). Hence, it is possible that prolonged activation of sympathetic neurons results in the release of DA from nerve terminals of the sympathetic neurons.

DOPA was reported to be a substrate of L-type amino acid transporter (LAT) 1 and 2 (43, 44). The reduced expression of LAT2 by siRNA in proximal tubule cultured cell decreased the uptake of DOPA (45), and LAT2 overexpression increased the uptake of DOPA (46). These data suggest that the DOPA contents in the cells are regulated by the LAT1/LAT2 activity. In our experiments, no correlation was observed between the DOPA content in the plasma and that in the tissues, except for the kidney. In the kidney, both DA and DOPA concentrations were correlated with plasma DOPA concentration (Fig. 7, *L* and *R*; *r* = 0.629 and 0.686, respectively). Therefore, these data suggest that kidney takes up circulating DOPA and utilizes it for the DA synthesis.

As we detected TH and AADC immunoreactivity in airway epithelial cells (Fig. 8, *C* and *D*), they may produce DA by themselves. Because D1R expression in airway epithelial cells has been reported (28), DA function in these cells may be an autocrine signal. As DA synthesis in the lung was thought to be from circulating DOPA (8, 47), to our knowledge, this is the first report of DA synthetic pathway by TH and AADC in the lung.

D1R was reported to be expressed in proximal tubules of the kidney, airway epithelial cells of the lung, and mucosal cells of the stomach (10, 27). In contrast, we detected X-gal staining in the muscularis externa of the stomach and around the blood vessels of the lung (Fig. 8). The reason for the differences between our present results and the previous reports is not clear, and immunohistochemical and/or *in situ* hybridization analysis are required to localize the expression of D1R protein and mRNA.

In conclusion, we produced mice in which the *Th* gene is selectively ablated in the sympathoadrenal system and examined the tissue DA metabolism in peripheral tissues in detail. Our data unravel differences in the DA metabolism among tissues and suggest that some peripheral tissues have autonomous DA-producing ability. Our results also support the idea that sympathetic neurons may supply DA as well as NA to the peripheral organs. Further study will be required to demonstrate the release of DA from the sympathetic neurons.

## Experimental procedures

### Animals

Mice were housed at 25 °C under a 12-h light/dark cycle with water and chow provided *ad libitum*. All animal experiments were conducted in accordance with the Guidelines for the Care and Use of Laboratory Animals of Tokyo Institute of Technology (D2016003, D2019008). *Th*-floxed mice were previously described (48). To generate *DBH-CreERT2* transgenic mice, a DNA segment encompassing the −5.8 kb to +54 bp of the human DBH gene (24) was cloned into the pGS-CreERT2 vector (25) and the NotI restriction segment encompassing the transgene was microinjected into mice. The resulting DBH-CreERT2 transgenic mice were backcrossed on C57/BL6 mice for more than 10 generations and intercrossed with floxed TH mice, raised on a similar genetic background.

For the detection of *Th* recombined allele, genomic DNAs from the heart, lung, spleen, stomach, pancreas, kidney, adrenal gland, and superior cervical ganglion were extracted from Ctrl and cKO mice at 4 weeks after TAM injection. The recombined Th alleles were detected by PCR (94 °C for 30 s, 66 °C for 30 s, and 72 °C for 1 min 15 s for 27 cycles) with primers TH5F (5′-AGGCGTATCGCCAGCGCC-3′) and TH10Rb (5′-CCCCAGAGATGCAAGTC-CAATGTC-3′), as reported previously (48). The sizes of the PCR products from the floxed *Th* allele and recombined *Th* allele were 1886 and 430 bp, respectively.

TAM was prepared and injected according to a previously published method (25). Briefly, TAM was dissolved in ethanol and then diluted 5 times with sunflower oil to 10 mg/ml. Eight-to 12-week-old male mice were intraperitoneally injected with TAM (1 mg/day) for 5 consecutive days. Mice were sacrificed at 2 or 4 weeks after the last TAM injection.

D1R reporter mice were generated by crossing D1R-tTA mice with TRE-D1R/lacZ mice as previously described (30).

### CA measurement

Mice were deeply anesthetized with pentobarbital, and blood was collected from the right atrium with a heparinized syringe. Blood was centrifuged at 1000*g* for 20 min, and plasma was collected from the supernatant. The brain, liver, kidney, adrenal gland, pancreas, stomach, spleen, lung, and heart were collected. Plasma and tissues were frozen with liquid nitrogen and stored at −80 °C until analysis. Tissues were homogenized with PBS containing 0.25 mM EDTA, 0.1 mM pargyline (an inhibitor of monoamine oxidase), 1 μg/ml pepstatin, 1 μg/ml leupeptin, and 1 mM phenylmethylsulfonyl fluoride, and tissues were then centrifuged at 20,400*g* for 15 min. Purification of CAs was conducted as previously reported (49) with some modifications. Supernatants were deproteinized with 0.2 M perchloric acid containing 0.1 mM EDTA, incubated on ice for 20 min, and centrifuged at 20,400*g* for 20 min. Two hundred microliters of supernatant was neutralized by 50 μl of 1 M potassium carbonate and 200 μl of 0.2 M Tris–HCl (pH 8.5) followed by the addition of 5 mg of aluminum oxide, and 10 μM isoproterenol (10 μl) was used as an internal control. Aluminum oxide was washed twice with 0.02 M Tris–HCl (pH 8.5) and water. CAs were eluted with 0.16 M acetic acid containing 8 mM phosphoric acid, separated, and detected by HPLC equipped with a reversed-phase column (SC5-ODS; Eicom) and electrochemical detector (ECD700).

### Western blot analysis

Aliquots of the tissue homogenates containing 30 μg of protein (3 μg for adrenal gland) were subjected to SDS-polyacrylamide gel electrophoresis and blotted to polyvinylidene fluoride membranes. Membranes were incubated with the following primary antibodies: rabbit anti-TH antibody (1:5000; made in our laboratory), rabbit anti-AADC antiserum (1:2000; made in our laboratory), mouse anti-β-actin antibody (1:10,000; A5441; Sigma–Aldrich), and rabbit anti-GAPDH antibody (1:10,000; GTX100118; GeneTex). The following peroxidase-conjugated secondary antibodies were used: goat anti-rabbit IgG antibody (10,000; NA9340V, GE Healthcare) or sheep antimouse IgG antibody (1:10,000; NA9310V; GE Healthcare) for β-actin. Immunoreactivity of TH, β-actin, and GAPDH was visualized with chemiluminescence (Immobilon Western HRP Substrate; Merck Millipore) and cooled charge-coupled device cameras (Ez-Capture MG; ATTO). FIJI (50) was used for image editing and quantification of band intensity. The linearity of the band intensity and the amount of TH protein were verified by serial dilution of each organ (Fig. S2).

### Immunohistochemistry

Under deep anesthesia, mice were transcardially perfused with 4% paraformaldehyde. Frozen tissue sections were stained with rabbit anti-TH antibody (1:1000; made in our laboratory) and biotinylated secondary antibody (1:2000; BA-1000; Vector Laboratories). Immunoreactivity was visualized with avidin-biotinylated peroxidase complex (Vectastain ABC kit; Vector Laboratories). Images were captured by a microscope (Eclipse E800; Nikon) with a cooled charge-coupled device camera (VB-6010; Keyence). Slices of the adrenal gland were incubated with 5% normal swine serum followed by the following primary antibodies: mouse anti-TH antibody (1:1000; MAB5280; Merck Millipore) and rabbit anti-AADC antiserum (1:2000; made in our laboratory). The following secondary antibodies were used: Alexa 546-conjugated goat anti-rabbit IgG (1:500; A11010; Invitrogen) and Alexa 488-conjugated goat antimouse IgG (1:500; A11029; Invitrogen). Images were acquired using a confocal microscope (LSM780; Carl Zeiss). Z-stack images were acquired and projected to one image using the “Z project” function with the “Max Intensity” algorithm in FIJI.

### LacZ staining

Perfusion fixation was conducted on deeply anesthetized mice with PBS containing 1.5% glutaraldehyde and 0.8% paraformaldehyde. The brain, stomach, kidney, and lung were dissected and incubated with X-gal staining solution containing 5 mM potassium ferrocyanide, 5 mM potassium ferricyanide, 2 mM magnesium chloride, and 0.5 mg/ml X-gal in PBS for 90 min at 37 °C. The reaction was stopped by adding 4% paraformaldehyde, and then frozen sections were generated. Nuclei were stained with hematoxylin solution (Fujifilm-Wako).

### Statistics

Data are shown as individual data and mean ± SEM. *p* Values less than 0.05 were considered statistically significant. Multiple comparisons were performed by Welch's *t* test corrected with Holm's method or two-way ANOVA followed by Tukey–Kramer test as a post hoc test. The correlation coefficient was calculated by Pearson's test. Statistical analyses were conducted by R (51), version 3.6.2.

## Data availability

All data from this study are contained within the article, including supporting information.

## Supporting information

This article contains supporting information.

## Conflict of interest

The authors declare that they have no conflicts of interest with the contents of this article.
