# Supplementary material for: Tyrosine hydroxylase conditional KO mice reveal peripheral tissue-dependent differences in dopamine biosynthetic pathways
Source: J Biol Chem. 2021 Mar 15;296:100544. doi: 10.1016/j.jbc.2021.100544 (PMC8076703; doi:10.1016/j.jbc.2021.100544)
Supplement: Figures S1 and S2 [file mmc1.pdf]

Supporting Information

Tyrosine hydroxylase conditional knockout mice reveal peripheral tissue-dependent differences in dopamine biosynthetic pathways

Katsuya Miyajima, Chiaki Kawamoto, Satoshi Hara, Masayo Mori-Kojima, Tamae Ohye, Chiho Sumi-Ichinose, Nae Saito, Toshikuni Sasaoka, Daniel Metzger, Hiroshi Ichinose

Figure S1. Confirmation of inducible and selective ablation of the *Th* gene in the sympathoadrenal system after tamoxifen injection.

Figure S2. Non-cropped images of the Western blot analyses and the linearity of the quantitation of the protein bands.

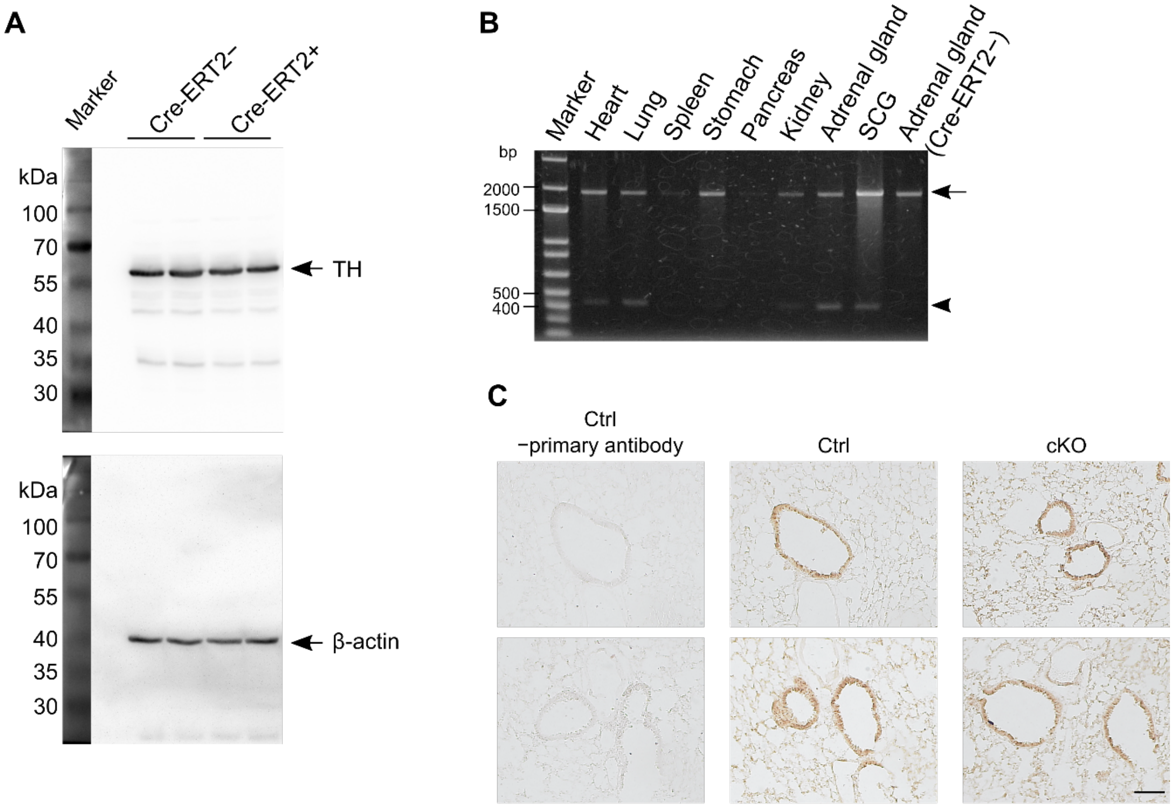

Figure S1. Confirmation of inducible and selective ablation of the *Th* gene in the sympathoadrenal system after tamoxifen injection. ***A,*** Western blot analysis in the adrenal gland both of Ctrl and cKO mice prior to tamoxifen induction. The adrenal glands of *Th*$^{\mathrm{fl/fl}}$ mice (CreERT2–) and *Th*$^{\mathrm{fl/fl}}$ with *DBH Cre-ERT2* mice (CreERT2+) were subjected to western blot analysis prior to tamoxifen induction. The TH band intensity from CreERT2+ mice was comparable to those from CreERT2– mice, indicating little or no leaking spontaneous recombination in the *Th* gene. *B,* PCR detection of the *Th* gene recombination. Genomic DNA was extracted from heart, lung, spleen, stomach, pancreas, kidney, adrenal gland, and superior cervical ganglion (SCG) of cKO (CreERT2+) and Ctrl (CreERT2–) mice at 4 weeks after tamoxifen injection. The recombined *Th* alleles, indicated by an arrowhead, were detected in the DNAs extracted from the heart, lung, adrenal gland and SCG. *C,* Immunohistochemical analysis of TH in the lung of *Th*$^{\mathrm{fl/fl}}$ mouse (Ctrl) and *Th*$^{\mathrm{fl/fl}}$ with *DBH Cre-ERT2* mouse (cKO) at 4 weeks after tamoxifen injection. Note that TH immunoreactivity was not affected in the lung of cKO mouse after tamoxifen injection. Scale bar, 100 μm.

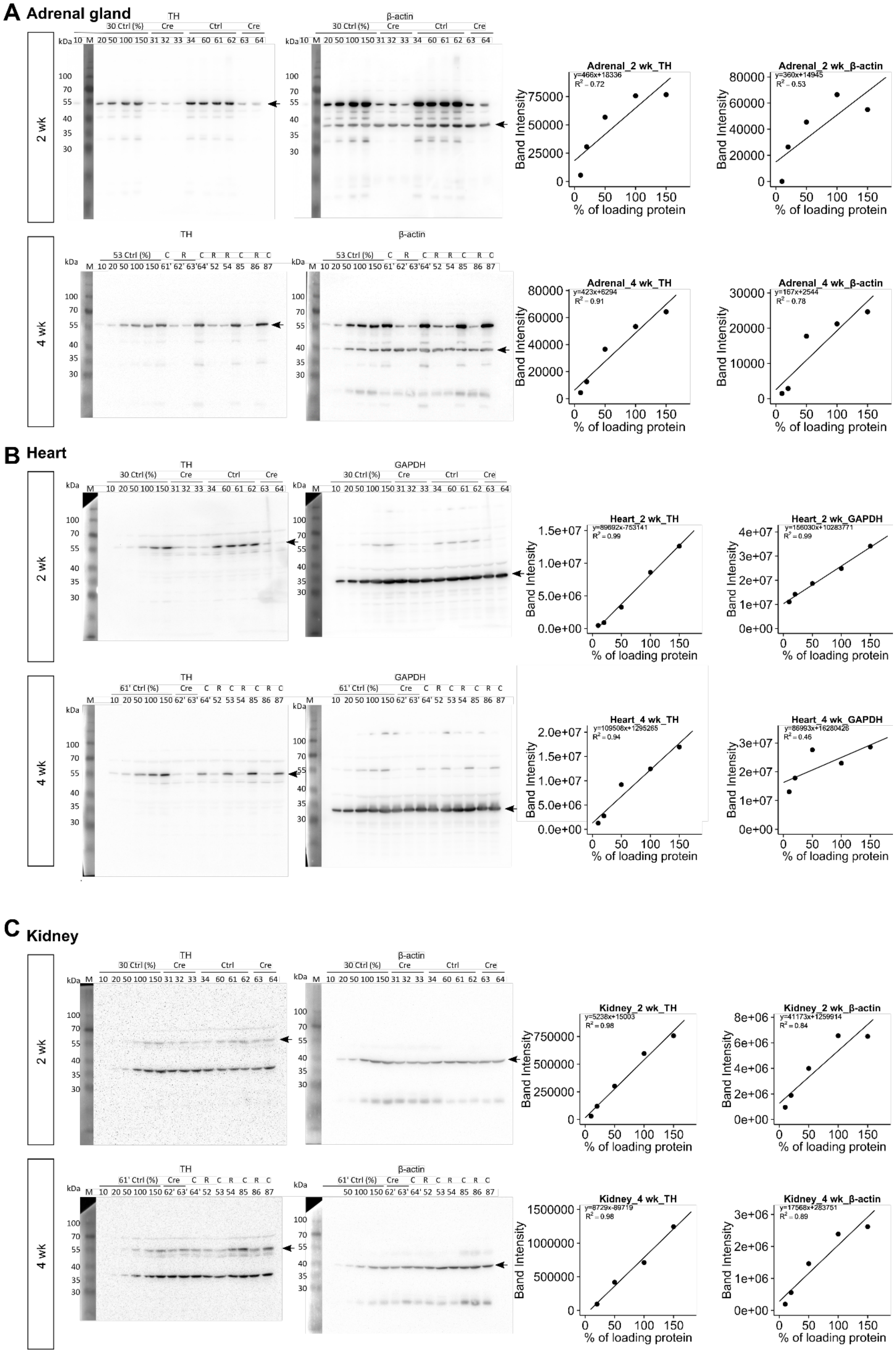

Figure. S2 (Figure continues on the next page)

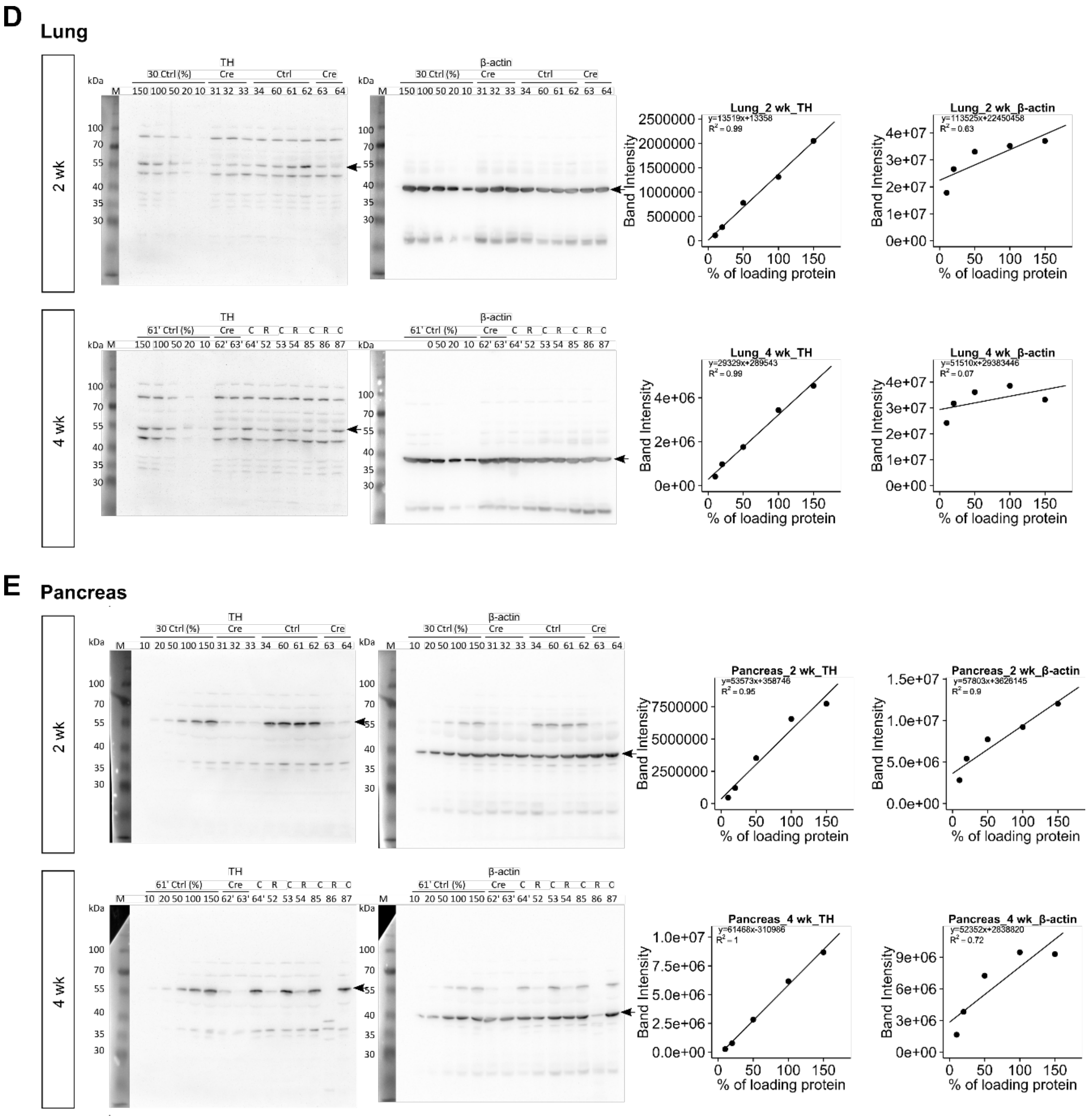

Figure. S2 (Figure continues on the next page)

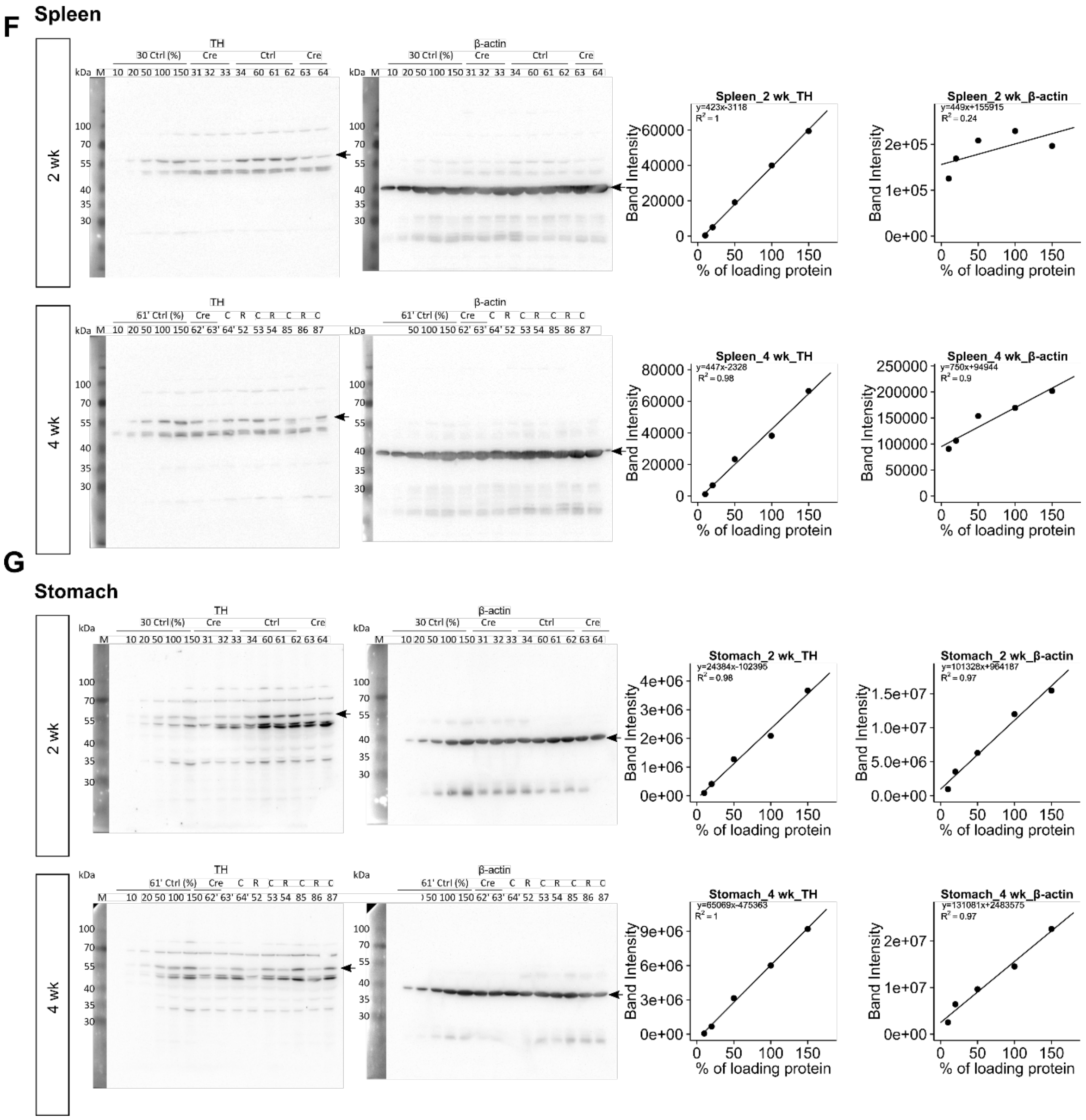

Figure S2 Non-cropped images of the Western blot analyses and the linearity of the quantitation of the protein bands. Whole membrane images and examining the linearity of band intensity. The tissues of *Th*$^{fl/fl}$ (Ctrl or C) and *Th*$^{fl/fl}$ with *DBH Cre-ERT2* mice (Cre or R) were prepared for Western blot analysis at 2 or 4 weeks after tamoxifen injection (2wk and 4 wk, respectively). Linearities were checked by serial dilution (10, 20, 50, 100, and 150% of protein) of Ctrl sample, and linearities were visualized in the right graphs. A adrenal gland, B heart, C, kidney, D, lung, E, pancreas, F, spleen, and G, stomach.
